# Collaborative Management of Patients With Estrogen Receptor–Positive Breast Cancer

**Published:** 2017-04-01

**Authors:** Lee S. Schwartzberg, Heather R. Greene

**Affiliations:** West Cancer Center, Memphis, Tennessee

## Abstract

Cyclin-dependent kinase (CDK) inhibitors represent a new form of cytotoxic chemotherapy. Advanced practitioners can read this article to find out about the science behind CDK inhibition, when CDKs are indicated, how to monitor for and manage side effects, as well as when and how to dose adjust.

Although remarkable progress has been made in the treatment of early-stage breast cancer, about 20% of women will relapse from an original diagnosis of stages I–III breast cancer and develop metastatic breast cancer (MBC), according to Lee S. Schwartzberg, MD, FACP, of the West Cancer Center, Memphis, Tennessee. With the emergence of targeted therapies, including the important new cyclin-dependent kinase (CDK) 4/6 inhibitors for estrogen receptor (ER)–positive disease, some forms of MBC are evolving into chronic diseases.

In fact, "these patients now are living years to decades," added Dr. Schwartzberg, "and that is wonderful progress."

At the 2016 JADPRO Live conference, Dr. Schwartzberg was joined by Heather R. Greene, MSN, FNP, AOCNP®, also of West Cancer Center, Memphis, Tennessee, to discuss the clinical predictors of outcome for women with ER-positive breast cancer; the role of the CDK4/6 inhibitor palbociclib (Ibrance) in the treatment of this MBC subset; the side-effect profile of these agents; and strategies for managing toxicity while preserving dose delivery with these oral drugs.

## CLINICAL PREDICTORS OF OUTCOME

The disease-free interval is among the various clinical predictors of outcome for women with ER–positive breast cancer. "We have known for a long time now that the natural history of metastatic ER–positive breast cancer can be very prolonged. It is not surprising to see women relapse 10 to 15 years after their initial diagnosis," Dr. Schwartzberg said.

One clinical predictor of outcome is prior endocrine therapy, which Dr. Schwartzberg says has become especially important, with the widespread use of adjuvant therapy. "What happens to those women who have had years of adjuvant endocrine therapy when they relapse is very different from what happens to a woman who has never had adjuvant endocrine therapy," Dr. Schwartzberg indicated.

Next, quantitative ER expression is "a good predictive factor to response to hormone therapy," Dr. Schwartzberg noted (Rakha et al., 2007; Loi et al., 2008). For example, a patient who has a low level of ER expression is less likely to respond to therapy than a patient with high expression.

Two other clinical predictors of response include the presence of bone-only vs. visceral metastases (Niikura et al., 2011) and HER2 expression. Brain and visceral metastases, according to Dr. Schwartzberg, tend to be less common in women who have hormone receptor–positive disease, and this factor may influence how these women are treated. HER2 expression certainly guides the choice of therapies for these women, usually with chemotherapy and anti-HER2 agents.

## PALBOCICLIB IN BREAST CANCER: CLINICAL TRIAL RESULTS

With resistance to endocrine therapy representing a major clinical challenge in hormone receptor–positive breast cancer, the emergence of CDK4/6 inhibitors has been met with much enthusiasm. The growth of hormone receptor–positive breast cancer is dependent on cyclin D1, which is a direct transcriptional target of the estrogen receptor (Turner et al., 2015a).

Preclinical trials performed several years ago with the CDK4/6 inhibitor palbociclib showed it to be synergistic with tamoxifen and trastuzumab (Herceptin) in ER–positive and HER2-amplified cell lines, respectively (Finn et al., 2009). These findings led to the phase II PALOMA-1 clinical trial (Finn et al., 2015), for which palbociclib received Breakthrough Therapy designation by the FDA. This open-label, randomized phase II trial included postmenopausal women with advanced ER–positive and HER2-negative breast cancer not previously treated for advanced disease.

Palbociclib in combination with the nonsteroidal aromatase inhibitor letrozole for first-line treatment proved effective, with about a 50% improvement in progression-free survival (PFS) over letrozole alone. "The curves separate very early," noted Dr. Schwartzberg, and "they remain separated out to 3-plus years of follow-up." Based on these phase II findings, the FDA granted palbociclib Accelerated Approval for metastatic ER–positive breast cancer in early 2015.

A follow-up phase III trial, comparing letrozole to letrozole and palbociclib in the first-line setting (PALOMA-2) was launched. In the most recent update of the PALOMA-2 trial findings (Finn et al., 2016), the improvement in PFS was 10 months, similar to PALOMA-1, and the clinical benefit rate was 85%, with advantages seen in all subgroups. Putting this into clinical perspective, Dr. Schwartzberg commented, "That means 5 out of 6 women benefited from the combination therapy, [which are] pretty staggering numbers when you treat metastatic disease with any type of therapy."

A phase III study, known as PALOMA-3, involved more than 500 women with advanced hormone receptor–positive, HER2-negative breast cancer who had relapsed or progressed during prior endocrine therapy. In this trial, palbociclib was combined with fulvestrant (Faslodex) and compared with fulvestrant alone (Turner et al., 2015a, 2015b). In what Dr. Schwartzberg referred to as "pretty striking results," the combination therapy yielded better PFS than fulvestrant alone (9.2 vs. 3.8 months), with a hazard ratio of 0.42 (95% confidence interval [CI], 0.32–0.56; *p* < .001).

Dr. Schwartzberg briefly focused on two subgroups: those with visceral metastases (60%) and women who were pre- or perimenopausal (20%). Responses were not compromised by the presence of visceral metastases or pre- or perimenopausal status. "This is the first study to show that you can actually treat pre-menopausal patients in the metastatic setting with ovarian function suppression and appropriate endocrine therapy, and see the same benefit as in post-menopausal patients," Dr. Schwartzberg revealed.

The final analysis of the PALOMA-3 trial (Cristofanilli et al., 2016) confirmed the significant and consistent improvement in PFS with the combination of palbociclib and fulvestrant, regardless of the degree of endocrine resistance, hormone receptor expression level, and *PIK3CA* mutational status. Overall survival data are immature at this time and eagerly awaited, he said.

**Side-Effect Profile**

"The predominant downside of giving palbociclib is hematologic, particularly neutropenia," Dr. Schwartzberg indicated. Approximately 50% of patients on palbociclib plus fulvestrant developed grade 3 neutropenia, and 9% developed grade 4 neutropenia. Other hematologic adverse events included leukopenia, anemia, and thrombocytopenia. The incidence of serious adverse events was similar between the treatment groups. In addition, receipt of palbociclib also increased the risk of infections (34.2% vs. 24.4%), though the majority were grade 1 or 2. No deaths due to adverse events or toxicity were reported.

## STRATEGIES FOR MONITORING PATIENTS ON ORAL THERAPY

Ms. Greene offered her clinical experience and practical strategies to help women with breast cancer remain adherent to their oral regimen, particularly palbociclib, to reap the full benefits of therapy. To begin, she reviewed the recommended dosing for palbociclib (Pfizer, 2016): The starting dose is 125 mg/day for 21 days every 28 days.

Some side effects may require dose interruptions, delays, or modifications. The first dose reduction is to 100 mg/day, and the second dose reduction is to 75 mg/day. Ms. Greene stressed the importance of educating patients about the need to take this agent with food. "It has been my experience that if you have patients on two oral therapies (such as letrozole and palbociclib), especially older patients, remind them to take the letrozole every day and the palbociclib 21 days and then a break," she suggested. Although these regimens may not seem complicated to health-care professionals, they can be confusing to patients. Visual aids, such as calendars, can help patients remember their proper dosing schedule.

In addressing the toxicity associated with palbociclib, Ms. Greene stressed the hematologic side effects. Neutropenia is essentially universal, whether palbociclib is given with letrozole or fulvestrant. Advanced practitioners should check complete blood cell (CBC) counts on day 1 of every cycle. "And for at least cycles 1 and 2, we need to check their CBC on day 15," she advised.

Other side effects to watch for are fatigue, which can "certainly be an issue," and alopecia, she said. Hair loss that occurs with palbociclib is different from alopecia associated with cytotoxic chemotherapy. It is usually grade 1 (thinning, patchy), rather than the total alopecia often seen with cytotoxic chemotherapy.

**Using the CTCAE Grading Scale**

Advanced practitioners who are caring for patients on CDK4/6 inhibitors need to be familiar with the Common Terminology Criteria for Adverse Events (CTCAE) grading scale. "You need to know what a grade 2 hematologic toxicity is, so you can manage these patients appropriately," Ms. Greene said.

Different dose modifications are required for the different grades of hematologic toxicities associated with palbociclib. For a grade 1 or 2 toxicity, no change is necessary. For a grade 4 side toxicity, therapy should be held "no matter where patients are in their cycle," Ms. Greene stressed. When the toxicity drops to grade 2 or lower, therapy can be resumed at the next lower dose.

Grade 3 hematologic toxicities are more complicated. When the problem occurs on day 1 of the cycle, the dose should be held and the CBC count repeated in 1 week. If the toxicity drops to grade 2 or lower, the drug can be resumed at the same dose. However, if the toxicity is noticed on day 15 of either cycle 1 or 2, the current dose can be continued and a CBC count repeated on day 21. It is important that the toxicity ameliorates before the next cycle is initiated. Finally, for grade 3 neutropenia accompanied by a complication, such as a fever or infection, treatment should be held until the toxicity recovers to grade 2 or lower, with therapy resumed at the next-lower dose. Ms. Greene suggested that advanced practitioners use the measurements from the CTCAE for neutropenia grading (Table).

**Table:**
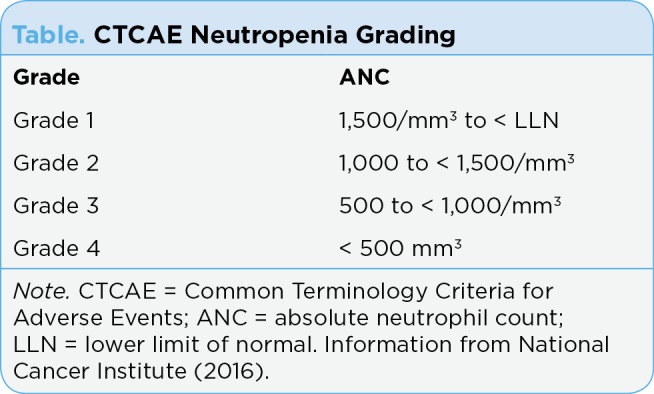
CTCAE Neutropenia Grading

For nonhematologic side effects such as diarrhea, dose modifications are not needed for grade 1 or 2 toxicities. However, for patients with ≥ grade 3 diarrhea that continues despite dietary changes and the use of loperamide, the dose should be held until diarrhea returns to grade 1 or less; if it returns to grade 2 or lower (with no safety risks), the next-lower dose can be resumed (Pfizer, 2016).

It is also important for advanced practitioners to be familiar with potential drug interactions. For instance, the use of CYP3A inhibitors such as clarithromycin, grapefruit, and ketoconazole can increase palbociclib levels (Pfizer, 2016). "You need to review the patient’s medications, and if you are unfamiliar with what might be an interaction, talk with your oncology pharmacist," she advised. In addition, patients who are taking CYP3A inducers such as carbamazepine, phenytoin, or St. John’s wort will need to "switch to a safer alternative" while taking palbociclib; palbociclib levels will be decreased up to 85% with these CYP3A inducers (Pfizer, 2016).

## OTHER CDK4/6 INHIBITORS

Dr. Schwartzberg and Ms. Greene briefly mentioned two other CDK4/6 inhibitors that are in late-stage clinical investigation—ribociclib (formerly known as LEE011) and abemaciclib. In the preclinical NVP-LEE01 trial, ribociclib appeared to be preferentially active in ER–positive breast cancer cell lines, according to Dr. Schwartzberg.

In November 2016, the FDA granted Priority Review for ribociclib as first-line treatment of postmenopausal women with hormone receptor–positive, HER2-negative MBC in combination with letrozole. This ruling was based on the results of the phase III MONALEESA-2 trial (Hortobagyi et al., 2016). MONALEESA-2 met its primary endpoint early, showing a significant improvement in PFS, though rates of myelosuppression were higher with ribociclib, Dr. Schwartzberg indicated. Ribociclib was approved by the FDA in March of 2017.

In the phase I MONARCH 1 trial of abemaciclib (Dickler et al., 2016), the investigational agent induced objective tumor responses as monotherapy in some patients with refractory hormone receptor–positive MBC following multiple prior therapies. "Interestingly, neutropenia was not the dose-limiting toxicity," Dr. Schwartzberg noted, "and continuous dosing was feasible." Phase III trial results of abemaciclib in combination with a nonsteroidal aromatase inhibitor are expected in the near future.

In closing, Dr. Schwartzberg offered his clinical perspective on the emergence of CDK4/6 inhibitors such as palbociclib: "The uptake in our clinic for using this agent [palbociclib] and in my own practice as a medical oncologist has been pretty extraordinary for a new drug. The large majority of patients who have first-line metastatic, hormone receptor–positive breast cancer are getting combination therapy."
